# Non-Invasive Myocardial Work Identifies Patients with Obstructive Coronary Lesions After Orthotopic Heart Transplantation

**DOI:** 10.3390/diagnostics15111352

**Published:** 2025-05-28

**Authors:** Rebeca Manrique Antón, Marina Pascual Izco, Gorka Bastarrika, Agnés Díaz Dorronsoro, Ana Ezponda, Fátima de la Torre Carazo, Nahikari Salteráin, Leticia Jimeno-San Martín, Nerea Martín-Calvo, María Josefa Iribarren, Gregorio Rábago

**Affiliations:** 1 Department of Cardiology and Cardiac Surgery, Clínica Universidad de Navarra, University of Navarra and School of Medicine, Av. De Pío XII 36, 31007 Pamplona, Spain; mpascualiz@unav.es (M.P.I.); adiazdo@unav.es (A.D.D.); nsalterain@unav.es (N.S.); ljimeno@unav.es (L.J.-S.M.); grabago@unav.es (G.R.); 2 Instituto de Investigación Sanitaria de Navarra (IdiSNA), 31008 Pamplona, Spain; bastarrika@unav.es (G.B.); aezponda@unav.es (A.E.); 3 Department of Radiology, Clínica Universidad de Navarra, University of Navarra and School of Medicine, Av. De Pío XII 36, 31007 Pamplona, Spain; 4 Department of Cardiology, Hospital Universitario Ramón y Cajal, 28034 Madrid, Spain; fdelatorrec@gmail.com; 5 Department of Preventive Medicine and Public Health, University of Navarra and School of Medicine, 31008 Pamplona, Spain; nmartincalvo@unav.es; 6 Department of Anesthesia and Critical Care, Clínica Universidad de Navarra, University of Navarra and School of Medicine, Av. De Pío XII 36, 31007 Pamplona, Spain; mjiriba@unav.es

**Keywords:** heart transplantation, cardiac allograft vasculopathy, speckle-tracking, strain, myocardial work, CT

## Abstract

**Background/Objectives:** Cardiac allograft vasculopathy (CAV) is a major complication following orthotopic heart transplantation (OHT). Graft denervation results in silent ischemia, even when already established, requiring regular screening for early diagnosis. This study explores whether myocardial work (MW) can non-invasively identify OHT patients with obstructive coronary lesions (OCL). **Methods**: During regular follow-ups, 55 OHT recipients underwent paired, prospective coronary computed tomography angiography (CCTA) and transthoracic echocardiography (TTE) examinations. Additionally, 57 healthy volunteers (HV) provided reference TTE data. Classic echocardiographic parameters, such as left ventricle global longitudinal strain (LV-GLS) and MW indices, were obtained in all individuals. Data from three groups were analyzed: HV, OHT patients without coronary lesions or with <50% lesions on the CCTA (OHT-non-OCL), and OHT patients with ≥50% lesions on the CCTA (OHT-OCL). **Results**: CCTA identified seven OHT patients with OCL. Significant differences across the groups existed for LV-GLS (OHT-OCL −10.6% CI −14 to −6.8 vs. OHT-non-OCL −15.6% CI −16.5 to −13.4% vs. HV −18% CI −20 to −16, *p* < 0.01) and global work efficiency (GWE) (OHT-OCL 87% CI 86 to 92 vs. OHT-non-OCL 94% CI 91 to 95 vs. HV 96% CI 95 to 97, *p* < 0.01). The optimal cut-off values identified using the Youden Index were LV-GLS < −14.4% (AUC 0.80, sensitivity 0.86, specificity 0.71) and GWE < 89% (AUC 0.75, sensitivity 0.71, specificity 0.85). Multivariate analysis showed GWE as the best marker for detecting OCL. **Conclusions**: GWE is the echocardiographic parameter that best identifies OHT patients that have OCL on CCTA. If validated in larger studies, GWE could become a readily accessible tool for CAV detection.

## 1. Introduction

Cardiac allograft vasculopathy (CAV) is a common complication following orthotopic heart transplant (OHT), and is a major cause of graft loss and mortality [1]. Histopathologically, CAV manifests as a diffuse intimal thickening, leading to allograft ischemia. Despite this, patients often remain asymptomatic due to graft denervation [2,3,4,5]. For this reason, the International Society for Heart and Lung Transplantation (ISHLT) recommends performing annual or biannual invasive coronary angiography (ICA) to monitor the development of CAV, and to use a standardized nomenclature for grading angiographic findings related to CAV. Baseline intravascular ultrasound (IVUS) or optical coherence tomography (OCT), combined with ICA at 4 to 6 weeks and 1 year post-heart transplantation, is recommended to detect donor-transmitted or identify rapidly progressing cardiac allograft vasculopathy [6], but these are invasive and costly procedures [7].

Non-invasive imaging techniques are employed for CAV surveillance, with reasonable diagnostic performance in detecting angiographic stenosis of 50% or greater. However, heir sensitivity diminishes when identifying less severe cases, such as ISHLT grade CAV1, early coronary intimal thickening, and microvascular disease. Due to the lack of robust diagnostic and prognostic evidence supporting any single modality, the choice of non-invasive surveillance largely depends on the expertise available at each center. Coronary computed tomography angiography (CCTA) may be used as a noninvasive alternative to ICA for the detection of CAV in ≥2 mm epicardial vessels [6].

In some centers, CCTA has been implemented as the primary tool for CAV surveillance [8,9], reserving ICA for cases where CCTA results are inconclusive or indicate significant disease. This approach aligns with ISHLT recommendations and reflects a growing trend toward adopting non-invasive techniques in clinical practice.

Although CCTA serves as a non-invasive alternative for detecting CAV [8], it requires intravenous contrast and exposes patients to ionizing radiation. These concerns are heightened in OHT patients with impaired renal function and those undergoing frequent imaging, leading to cumulative radiation exposure [7].

Non-invasive, non-irradiating methods like advanced echocardiography are gaining interest for CAV detection [7]. Left ventricle global longitudinal strain (LV-GLS) has been linked to CAV severity [10,11], and is the most validated, feasible, and reproducible myocardial deformation parameter in clinical practice. Compared with radial and circumferential strains, GLS offers greater consistency and lower variability [12], although all strain components can be influenced by loading conditions.

Myocardial work (MW) is an innovative method that evaluates LV function through pressure–strain loop analysis, incorporating non-invasive arterial pressure to provide deeper insights into myocardial performance [13]. Although studies have shown its effectiveness in detecting coronary disease in the general population [14,15,16,17], its clinical utility in identifying this condition in adult OHT patients is not well established.

The primary objective of this study was to evaluate the utility of MW in discriminating between OHT patients with and without obstructive coronary lesions (OCL). The secondary goal was to identify the most effective MW parameter for detecting these lesions. Finally, the study also aimed to assess the applicability of normality cut-off values for MW to OHT patients.

## 2. Materials and Methods

### 2.1. Study Subjects

CCTA and transthoracic echocardiography (TTE) were prospectively conducted on 55 OHT recipients, with TTE performed 2–3 h post-CCTA, or, rarely, on consecutive days. Patients underwent OHT and received biannual follow-ups at our institution; they were consecutively enrolled between March 2021 and March 2023. The study design excluded individuals with previous incomplete percutaneous revascularization, diffuse narrowing or small vessel disease, along with those exhibiting ischemic symptoms, positive stress TTE for ischemia, suboptimal CCTA quality due to arrhythmias (*n* = 1), or inadequate acoustic window in TTE (*n* = 2). Patients with prior coronary intervention in the transplanted heart with favorable angiographic results were included. Additionally, 57 healthy volunteers (HV) with similar age and gender distribution to the OHT group were prospectively enrolled to provide reference TTE data. The study adhered to the Declaration of Helsinki and was approved by the local Ethics Committee. All participants provided written informed consent.

### 2.2. Demographic and Clinical Data

Demographic information (age, gender, and body mass index) and cardiovascular risk factors (hypertension, hyperlipidemia, diabetes mellitus, and smoking history) were systematically collected. Additionally, clinical details specific to OHT recipients (estimated glomerular filtration rate or eGFR, hemoglobin, cytomegalovirus infection, presence of cardiac devices, and peripheral artery disease) and OHT-related factors (age at OHT, retransplant status, years since OHT, etiology of the primary cardiomyopathy, ABO blood group, emergent or elective code, bridge-to-transplant ECMO, ischemic time, and post-transplant ECMO use) were documented. Pre-existing HLA antibodies were not included in the study due to lack of early-year register data. Furthermore, information on the administration of relevant medications (immunosuppressive drugs, antiplatelet agents, and lipid-lowering treatments) was also registered.

### 2.3. Two-Dimensional TTE

All OHT patients and HV underwent TTE using a Vivid™ E95 4D system equipped with an M5S 3.5 MHz transducer (GE Healthcare, Milwaukee, WI, USA), with individuals scanned in the left lateral position. Standard 2D images were saved in digital cineloop format for offline analysis. All measurements were performed by one expert echocardiographer, who was blinded to the CT scan findings.

From a parasternal view, 2D measurements included LV septal (IVSd) and posterior wall thickness (PWd), and end-diastolic diameter (LVDd). The LV mass index was calculated as the anatomic mass divided by body surface area, and relative wall thickness (RWT) with the formula (2 × PWd)/(LVDd). From an apical perspective, 2D LV ejection fraction (LV-EF) measurements were based on end-systolic/diastolic volumes using the disk biplane technique. For the analysis of diastolic function, peak early (E) and late (A) diastolic transmitral flow velocity and E/A ratio, deceleration time (DT) of E wave, pulsed wave Tissue Doppler diastolic (E′) velocities at both septal and lateral mitral annulus, E/E′ ratio as an estimate of LV filling pressure, and isovolumetric relaxation time (IRT) were assessed. All 2D and Doppler recordings and measurements were conducted in accordance with European and American Guidelines [18].

To calculate LV-GLS, 2D images from the apical four-chamber, two-chamber, and three-chamber views were acquired. LV-GLS was quantified using semiautomated function imaging from vendor-specific offline analysis software (EchoPAC version 202; GE Healthcare, Milwaukee, WI, USA), with the region of interest determined using automated function imaging with manual correction. LV-GLS was calculated as the average of the segments using the 17-segment model.

**Figure 1:**
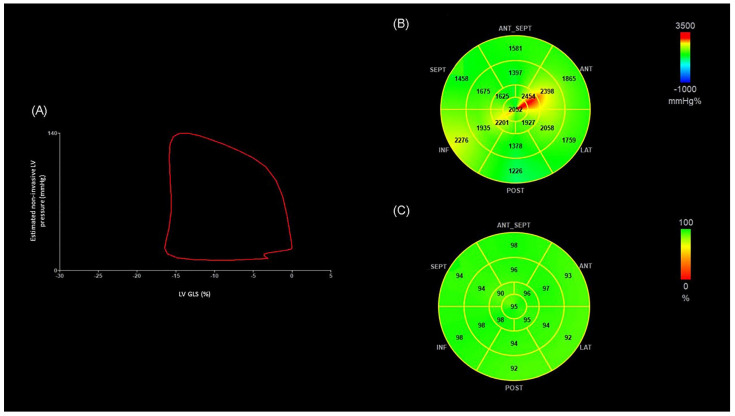
(**A**) Non-invasive LV pressure–strain loop diagram from an OHT patient without OCL. The LV pressure–strain loop exhibits a counterclockwise direction, with MWI corresponding to the area within the loop. (**B**) Segmental bull’s-eye MWI plots from the same OHT patient. Green, yellow, and red-coded areas on the plots indicate normal values. (**C**) Segmental bull’s-eye GWE plot from the same OHT patient. Green-coded areas on the plots indicate normal GWE. (LV-GLS −16%, MWI 1851 mmHg%, GWE 95%). ANT: anterior; ANT_SEPT: anteroseptal; GWE: global work efficiency; INF: inferior; LAT: lateral; LV-GLS: global longitudinal strain of left ventricle; LV: left ventricle, MWI: myocardial work index; POST: posterior; SEPT: septal.

MW indices were calculated by the same vendor-specific module (EchoPAC version 202; GE Healthcare, Milwaukee, WI, USA) combining the previously obtained LV strain data and a noninvasively estimated LV-pressure curve. Blood pressure (BP) was measured immediately before TTE, with peak systolic LV pressure assumed to be equal to peak arterial pressure. A non-invasive LV pressure curve was then constructed by the software according to the duration of isovolumic and ejection phases defined by the timing of aortic and mitral valve opening and closing events on 2D echocardiography. This estimated LV pressure curve was combined with strain to produce a LV pressure–strain loop (Figure 1), from which the software automatically calculated the following MW parameters [17]:

- Myocardial work index (MWI): the indexed total work performed by the LV during the systole, corresponding to the area of the loop.
- Global constructed work (GCW): the energy consumed by the myocardium contributing effectively to cardiac output (CO).
- Global wasted work (GWW): the myocardial work that does not contribute to CO.
- Global work efficiency (GWE): the ratio between GCW and the total (constructive and waste) work, reflecting the percentage of MW translating into CO [17,19].

### 2.4. CCTA Protocol and Study Analysis

CCTA examinations were performed on a third-generation dual-source CT scanner (SOMATOM Force, Siemens Healthineers) using the retrospectively ECG-gated acquisition mode with full tube current administration between 35% and 70% of the R-R’ interval and after biphasic intravenous injection of 70 mL of 300 mg I/mL iodinated contrast medium (iohexol, Omnipaque, GE Healthcare), followed by 50 mL of saline flush at 5 mL/s. Two minutes before the examination, all patients received 0.4 mg of sublingual nitroglycerine (Vernies, Pfizer).

Data were reconstructed with 0.6 mm slice thickness and 0.4 mm reconstruction increments using an iterative reconstruction technique (ADMIRE, Siemens Healthineers), with soft-tissue convolution kernel (Bv40), and strength level of 3. All images were stored in our institutional PACS.

CCTA studies were analyzed using dedicated cardiac postprocessing software (Syngo.via, Siemens Healthineers) on a per-segment basis following the 16-segment classification of the American Heart Association [20]. Degree of coronary stenosis was assessed visually by consensus by two experienced radiologists and graded as follows: 0%, 1–24%, 25–49%, 50–69%, 70–99%, or 100% stenosis or occlusion. CCTA studies were then classified into two groups based on the presence of nonobstructive CAV (defined as a lumen reduction of <50%) or obstructive disease (defined as a lumen reduction ≥ 50%). Coronary segments with a diameter <1.5 mm were excluded from the analysis.

### 2.5. Statistical Analysis

The normal distribution of variables was assessed with the skewness and kurtosis tests for normality. For descriptive purposes, means and standard deviations or medians and interquartile range were used for quantitative variables, and percentages for categorical variables. Comparisons between groups were performed with the Mann–Whitney U test (comparison of 2 groups) or the Kruskal–Wallis test (comparison of 3 groups). Categorical variables were compared using Fisher’s exact test. The area under the Receiver operating characteristic curve (AUC) of each echocardiographic variable was calculated using binary logistic regression models with OCL as the dependent variable. All continuous variables were dichotomized using the Youden Index (i.e., the maximum value for Sensitivity + Specificity −1) and their sensitivity and specificity for the diagnosis of OCL were calculated. Dichotomized echocardiographic variables were used to predict the odds ratio (OR) and 95% Confidence Interval (CI) of OCL using simple logistic regression models. The variables significantly associated in the univariate analysis were introduced in a forward stepwise model for multivariate analysis. All the analyses were performed with Stata 15.1. Statistical significance was settled at two-sided *p* value <0.05. Outlier values were not excluded from the analyses due to the limited sample size.

## 3. Results

### 3.1. Demographics

A group of 55 consecutive OHT patients and 57 HV were recruited. All OHT patients underwent both a CTTA and a TTE, while HV only underwent a TTE. Independent experts, blinded to the results of other tests and clinical data assessed TTE and CCTA images. CCTA identified OCL in seven OHT patients.

To determine which echocardiographic variables could identify patients with obstructive lesions, data from three groups were analyzed: healthy volunteers (HV), OHT without OCL (OHT-non-OCL), and OHT patients with OCL (OHT-OCL).

Demographic and clinical data of the three groups are summarized in Table 1. Baseline characteristics did not differ significantly, except for the prevalence of cardiovascular risk factors, such as hypertension, dyslipidemia, and diabetes (all *p* < 0.01).

A comparison of demographic and clinical characteristics between the two groups of OHT patients is provided in the Supplementary Materials (Table S1). Statistically significant differences were observed in several variables. The OHT-OCL group had a higher prevalence of diabetes mellitus (*p* < 0.01) and lower eGFR (*p* = 0.029). Furthermore, mycophenolate mofetil usage was higher in the OHT-non-OCL group (*p* < 0.01), whereas everolimus (*p* < 0.01) and ezetimibe usage was higher in the OHT-OCL group (*p* < 0.01).

### 3.2. Echocardiography Data

The conventional echocardiographic variables are summarized in Table 2. The Kruskal–Wallis test revealed significant differences among the three groups—particularly increased IVSd, PWd, and LV mass, as well as shorter IRT and E-wave DT in transplant recipients. These alterations were most evident in the OHT-OCL subgroup.

The advanced echocardiographic parameters detailed in Table 3 show significant overall differences among the three groups. These include LV-GLS (*p* < 0.01) and all MW parameters (*p* < 0.01), reflecting impaired myocardial performance in OHT-no-OCL patients, which is even more pronounced in the OHT-OCL group.

**Figure 2:**
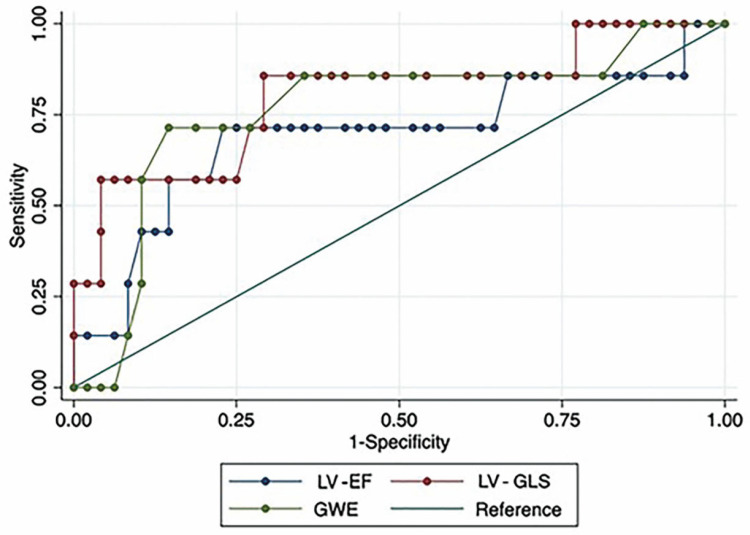
Receiver operating characteristic (ROC) curves for LV-EF, LV-GLS, and GWE. LV-EF: left ventricular ejection fraction; LV-GLS: global longitudinal strain of left ventricle; GWE: global work efficiency.

Both conventional and advanced echocardiographic parameters were dichotomized using the Youden Index, and the results are provided in the Supplementary Materials (Table S2). The resulting sensitivity and specificity for diagnosing OCL are presented in Table 4 and Figure 2. LV-EF < 60.6% had a sensitivity of 0.71 and a specificity of 0.77, resulting in an AUC of 0.69. Among advanced echocardiographic variables, LV-GLS <−14.4% showed a sensitivity of 0.86 and a specificity 0.71 with an AUC of 0.80, while GWE <89% had a sensitivity of 0.71 and specificity of 0.85, with an AUC of 0.75.

In the forward stepwise analysis, that included all the parameters associated with OCL in the univariate analysis (i.e., GWE, LV-GLS, LV-EF, GWW, EDS, LV mass index, and E wave), only LV-EF and GWE resulted as independent predictors of OCL (see Table 5).

Compared to patients with normal GWE, those with an abnormal value (defined after the Youden index) showed 15.65 times higher odds of having OCL (*p* = 0.008). Similarly, patients with LV-EF values below the cut-off value calculated with the Youden Index exhibited 9.08-fold higher odds of having OCL (*p* = 0.034).

A post hoc power analysis was conducted (Table 6). Comparing OHT recipients without OCL to those with OCL yielded a power of 62% for GWE and 41% for LV-GLS. In contrast, when comparing HV to OHT recipients with OCL, the power substantially increased to 99% for GWE and 63% for LV-GLS.

**Figure 3:**
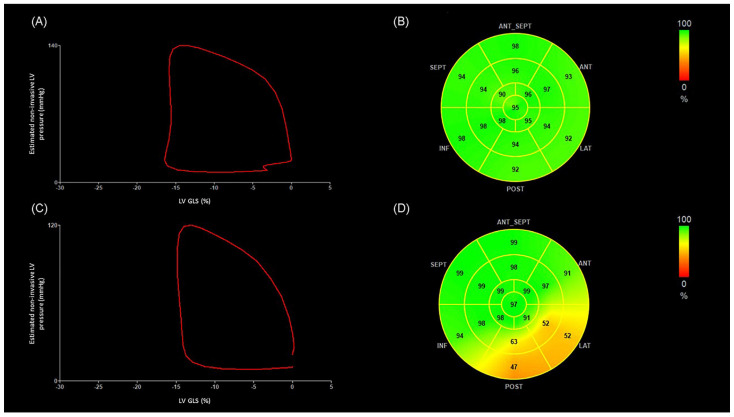
The figure illustrates representative images of LV pressure–strain loop diagrams and segmental bull’s-eye GWE plots of two OHT patients, one without OCL and the other with severe diffuse narrowing of circumflex artery. The area of the LV pressure–strain loop is visually smaller and the GWE values at the posterolateral segments are lower in the patient with OCL. (**A**) Non-invasive LV pressure–strain loop diagram from an OHT patient without OCL. The LV pressure–strain loop exhibits a counterclockwise direction, with MWI corresponding to the area within the loop. (**B**) Segmental bull’s-eye GWE plots from the same OHT patient. Green-coded areas on the plots indicate normal GWE. (GLS −16%, MWI 1851 mmHg%, GWE 95%). (**C**) Non-invasive LV pressure–strain loop diagram from an OHT patient presenting diffuse disease of the circumflex artery, which presents 70–99% stenosis in the proximal segment, 70–99% stenosis in the distal segment, and diffuse disease of the first obtuse marginal branch. (**D**) Segmental bull’s-eye GWE plot from the same OHT patient. Orange-coded areas on the plots indicate impaired GWE. (GLS −15%, MWI 1376 mmHg%, GWE 87%). ANT: anterior; ANT_SEPT: anteroseptal; GWE: global work efficiency; INF: inferior; LAT: lateral; LV-GLS: global longitudinal strain of left ventricle; LV: left ventricle; MWI: myocardial work index; OHT: orthotopic heart transplantation; POST: posterior; SEPT: septal.

Figure 3 depicts representative images of LV pressure–strain loop diagrams and segmental bull’s-eye GWE plots of two patients, one without OCL and the other with diffuse narrowing of circumflex artery. The area of the LV pressure–strain loop is visually smaller and the GWE values at the posterolateral segments are lower in the patient with OCL.

## 4. Discussion

To our knowledge, this is the first study to evaluate the effectiveness of non-invasive MW in detecting OCL in adult OHT patients. The multivariate analysis identified GWE and LV-EF as independent predictors of OCL, with GWE demonstrating the strongest association.

Our primary aim was to non-invasively identify patients with ≥50% coronary lesions, according to the recommendation of the IHLTS for non-invasive modalities [6], regardless of the severity classification of their CAV.

Diagnosis and grading of CAV rely on various factors, including coronary anatomy and cardiac function, as outlined by the ISHLT consensus statement. Severe CAV is defined by any degree of coronary artery disease, a LV-EF ≤ 45%, and/or evidence of restrictive physiology [21]. However, it is important to note that a normal LV-EF does not definitively exclude CAV presence [11,22,23]. In our study, patients with OCL had LV-EF values within the normal range, with no significant differences between groups. The LV-EF AUC for detecting OCL was 0.69, with 0.71 sensitivity and 0.77 specificity. While LV-EF was independently associated with OCL presence in our multivariate analysis, a reduced LV-EF should be interpreted more as an indicator of advanced CAV and poorer prognosis, rather than as a reliable screening tool for CAV.

Diastolic function analysis showed shorter E wave DT, shorter IRT, and higher E/A ratios in both OHT groups compared to HV (all *p* < 0.05). However, stepwise analysis found no diastolic dysfunction variables to be independent predictors of OCL presence in OHT patients, indicating their limited predictive value.

There is a trend towards integrating advanced echocardiographic methodologies into OHT monitoring, supported by European Association of Cardiovascular Imaging recommendations since 2015 [7]. Previous studies have largely focused on the utility of strain in OHT assessment. For instance, Saleh et al., in 2011 [24] established normal strain values in 40 adult OHT recipients without significant complications, contrasting them with those of 82 healthy individuals. They reported a mean LV-GLS of −13.43% ± 2.39% in OHT patients versus −17.28% ± 2.30% in controls (*p* < 0.01). Our findings align with theirs, as OHT-non-OCL exhibiting a mean LV-GLS value of −15.60% (CI −16.50% to −13.40%), contrasting with −18% (CI −20% to −16%) in HV (*p* < 0.01). 

Regarding the association between LV-GLS and CAV, Sciaccaluga et al., [22] studied 33 OHT patients, including 12 with CAV. They found that patients without CAV had higher LV-GLS values (−15.7% ± 3.4%) compared to those with CAV (−11.4% ± 1.9%, *p* < 0.01). Similarly, Clemmensen et al., [10] investigated 178 OHT patients and observed a decrease in LV-GLS values with increasing CAV severity (CAV 0 −16.7% ± 2.4%, CAV 1 −15.2% ± 2.9%; CAV 2–3 −14.0% ± 3.88%, *p* < 0.01). Consistent with these findings, our study revealed LV-GLS values of –15.60% (CI −16.50% to −13.40%) in OHT-non-OCL versus −10.60% (CI −14% to −6.80%) in OHT-OCL (*p* < 0.05). Using the Youden index, we established an LV-GLS value <−14.4% as the optimal cut-off for detecting OCL in OHT, with an AUC of 0.80 and sensitivity and specificity of 0.86 and 0.71, respectively. However, in our multivariate analysis, LV-GLS was not an independent predictor of OCL presence.

As previously noted, the main limitation of strain lies in its sensitivity to changes in afterload [25,26,27], which can lead to poorer strain values, potentially misrepresenting the LV contractile capacity. In contrast, MW integrates both strain and BP data, offering further understanding of myocardial performance. In our study, HV exhibited MW values comparable to those reported for individuals of the same age in the general population. Specifically, the MWI (1853 mmHg%, CI 1613 to 2064), GCW (2142 mmHg%, CI 1879 to 2328), GWW (59 mmHg%, CI 38 to 83), and GWE (96%, CI 95 to 97) observed in HV were similar to those previously reported for males and females in the general population (MWI 1866 ± 286 mmHg%, GCW 2226 ± 328 mmHg%, GWW 85 mmHg%, CI 49 to 129, GWE 96% CI 94 to 97 for males; MWI 2002 ± 270 mmHg%, GCW 2338 ± 386 mmHg%, GWW 90 mmHg% CI 48 to 145, GWE 95% CI 94 to 97 for females) [13].

Non-invasive MW has proven effective in detecting coronary artery disease in the general population [14,15,16,17]. However, the existing literature lacks descriptions of normal values for non-invasive MW of the LV in adult OHT populations.

Recently, Pradhan et al., [28] conducted a study examining the correlation between MW and CAV in 24 pediatric OHT recipients, 6 of whom had CAV, and 24 controls. They discovered that pediatric OHT patients without CAV exhibited impaired MW values (MWI 1426.22 mmHg%, GCW 1656.55 mmHg%, GWW 170.20 mmHg% and GWE 90.87%) compared to controls (MWI 1802.81 mmHg%, GCW 2007.43 mmHg%, GWW 81.47 mmHg% and GWE 95.41%). Additionally, OHT patients with CAV had significantly lower values of GWE (83.87%) compared to those without CAV (90.87%) and controls (95.41%). This suggests that GWE could be a valuable tool for CAV screening in this population. In the same line, our study revealed a more pronounced reduction in GWE among OHT-OCL (87% CI 86 to 92) compared to those OHT-non-OCL (94% CI 91 to 95) when using HV as reference (96% CI 95 to 97).

Furthermore, we established the optimal cut-off point for GWE at 89% to identify patients with OCL using the Youden index, achieving adequate sensitivity (0.71), specificity (0.85), and AUC (0.75).

As previously mentioned, stepwise logistic regression analysis identified GWE as the variable more strongly associated with the presence of OCL in the OHT patient population. This underscores the robustness of GWE in discriminating between OHT with and without OCL. The superiority of GWE over LV-EF may be attributed to its ability to detect more subtle changes in ventricular performance.

These results are consistent with recent retrospective findings suggesting that GWE may be a reliable noninvasive marker of CAV in heart transplant recipients. In a single-center study including 93 patients, conducted by Cacioli et al., [28] GWE was significantly lower in patients with CAV (86 ± 7%) compared to those without (91 ± 4%, *p* < 0.001), and was independently associated with the presence of CAV (OR 0.86, 95% CI 0.77–0.94, *p* = 0.002). Notably, however, that study assessed CAV of any severity, while our analysis focused specifically on OCL, a more advanced and clinically significant form of CAV.

In addition, our prospective design incorporated CCTA as the diagnostic method, with echocardiographic and tomographic evaluations performed on the same day or within consecutive days, minimizing potential variability due to disease progression. In contrast, the retrospective study accepted either ICA or CCTA, and allowed up to 12 months between imaging assessments. Despite these methodological differences, both studies consistently identified GWE as the parameter most strongly associated with CAV, reinforcing its potential utility in the noninvasive evaluation of heart transplant recipients.

### Limitations

One important limitation of our study is the relatively small sample size, particularly the number of patients with OCL. To better contextualize our findings, we conducted a post hoc power analysis (Table 6). When comparing OHT recipients without OCL versus those with OCL, the power was 62% for GWE and 41% for LV-GLS. However, in the comparison between HV and OHT recipients with OCL, statistical power increased markedly to 99% for GWE and 63% for LV-GLS. Although the power is suboptimal, our ability to detect statistically significant differences in GWE despite a limited sample size supports the robustness of this finding. Conversely, the lack of significant differences in other echocardiographic parameters may partly reflect limited statistical power rather than a true absence of effect. This is a preliminary single center study with a reduced sample size, intended to explore the potential utility of MW indices in identifying OCL in OHT recipients. These results emphasize the need for future studies with larger cohorts to confirm and expand upon these observations.

Additionally, we employed CCTA as the primary method for CAV screening and categorizing OHT patients into those with OCL and those without OCL, instead of ICA, as this has been the standard protocol at our institution since the early 2000s.

Finally, the cross-sectional design of the present study limits tracking MW changes as coronary lesions develop over time or reversibility of abnormal myocardial work indices. A follow-up study might assess how changes in GWE over time correlate with progression from non-obstructive to obstructive disease, as well as prognostic impact on graft survival.

## 5. Conclusions

This is the first study designed to evaluate the utility of MW to detect obstructive coronary lesions in adult OHT patients. Given the novelty of our study, we believe that our findings provide valuable preliminary data that can serve as the basis for future research with expanded sample sizes.

GWE is the echocardiographic parameter more strongly associated with the presence of OCL in OHT patients. If validated in larger studies, GWE could be used as a non-invasive and readily accessible method to identify OHT patients at risk because of the presence of coronary lesions. Consequently, in clinical practice, abnormal GWE values might prompt additional testing, earlier invasive angiography, or adjustments in immunosuppressive or lipid-lowering therapy.

Both LV-GLS and MW parameters are significantly impaired in OHT patients, suggesting that the transplantation procedure itself negatively affects myocardial function. For this reason, commonly used cut-off values for these variables should not be applied in OHT patients.

## Figures and Tables

**Table 1:** Demographic and clinical characteristics of OHT-OCL patients, OHT-non-OCL patients, and HV.

	OHT-OCL (*n* = 7)	OHT-non-OCL (*n* = 48)	HV (*n* = 57)	*p*
Age, years	65.20 (56.33–71.76)	67.52 (54.89–73.48)	63.10 (55.66–68.74)	0.582
Sex, male	7 (100)	35 (72.92)	47 (82.46)	0.226
BMI, kg/m^2^	25.30 (21.60–29.80)	26.20 (22.90–27.85)	25.30 (24.20–27.77)	0.965
SBP, mmHg	126.14 (28.87)	126.15 (17.80)	130.86 (16.89)	0.322
DBP, mmHg	80 (17.50)	80.50 (12.29)	80.51 (9.85)	0.975
Hypertension	5 (71.43)	30 (62.50)	17 (29.82)	<0.01
Dyslipidemia	6 (85.71)	33 (68.75)	23 (40.35)	<0.01
Diabetes mellitus	5 (71.43)	8 (16.67)	3 (5.26)	<0.01
Tobaccouse Never Current Former	1 (14.29) 0 (0) 6 (85.71)	23 (48.94) 2 (4.26) 22 (46.81)	34 (61.82) 5 (9.09) 16 (29.09)	0.046

Sociodemographic characteristics of the three study groups, OHT patients with OCL, OHT patients without OCL, and HV, are shown. Values are presented as means and standard deviations for data that follow a normal distribution and as median and interquartile ranges for data not normally distributed. HV: healthy volunteers; DBP: diastolic blood pressure; BMI: body mass index; eGFR: estimated glomerular filtration rate; OHT-non-OCL: orthotopic heart transplant recipients without obstructive coronary lesions on coronary computed tomography angiography; OHT-OCL: orthotopic heart transplant recipients with obstructive coronary lesions on coronary computed tomography angiography; SBP: systolic blood pressure.

**Table 2:** Conventional echocardiographic parameters in OHT-OCL patients, OHT-non-OCL patients, and HV.

	OHT-OCL (*n* = 7)	OHT-non-OCL (*n* = 48)	HV (*n* = 57)	*p*
IVSd, mm	11.69 (1.73)	10.62 (2.24)	9.44 (1.85)	<0.01
PWd, mm	11 (10.70–12)	9.05 (8–10.95)	9.10 (8.30–9.90)	<0.01
LVDd, mm	43.71 (6.40)	43.77 (5.17)	44.46 (5.53)	0.65
LV mass index, gr/m^2^	98.10 (85–106.30)	84.45 (67.20–95.40)	73.10 (56.70–89.40)	0.01
RWT	0.53 (0.44–0.57)	0.44 (0.39–0.53)	0.42 (0.38–0.47)	0.129
Biplane EDV index, ml/m^2^	39.20 (24.30–43.50)	29.45 (25.35–37.70)	42 (36.30–51.10)	<0.01
Biplane ESV index, ml/m^2^	15.30 (10.30–21.50)	10 (7.13–12.55)	13.70 (10.40–17.90)	<0.01
LV-EF, %	58.80 (55.60–74)	68 (61.30–75)	67.40 (62.60–72.80)	0.191
E wave, cm/s	52 (48–76)	68 (58–82)	67 (55–82)	0.311
E wave DT, ms	170 (120–210)	171 (140–196)	200 (170–240)	<0.01
E/A	1.30 (0.95–1.43)	1.51 (1.27–1.71)	0.93 (0.77–1.30)	<0.01
e’ septal, cm/s	7 (5–7)	7 (6–8)	7 (7–9)	0.07
e’ lateral, cm/s	10 (3)	11 (3)	10 (3)	0.047
Mean E/e’	7.27 (6.50–8.44)	7.22 (6.10–9.23)	7.85 (6.40–9.40)	0.862
IRT, ms	86.14 (13.03)	88.63 (19.12)	101.25 (18.45)	<0.01

The echocardiographic findings for conventional parameters across the three study groups are shown. Values are presented as means and standard deviations for data that follow a normal distribution and as median and interquartile ranges for data not normally distributed. *p*-values refer to overall differences among the three groups. Biplane EDV index: left ventricular end-diastolic volume index; cm/s: centimeters per second; LV-EF: left ventricular ejection fraction; biplane ESV index: left ventricular end-systolic volume index; E wave DT: deceleration time of E-wave; E wave: early diastolic mitral inflow velocity; E/A: ratio of E-wave to A-wave; e’ lateral: tissue Doppler E’ velocity at the lateral annulus; e’ septal: tissue Doppler E’ velocity at the septal annulus; HV: healthy volunteers; IRT: isovolumetric relaxation time; IVSd: interventricular septum thickness in diastole; LV mass index: left ventricular mass index; LVDd: left ventricular end-diastolic dimension; mean E/e’: ratio of E-wave to tissue Doppler e’ velocity; ms: millisecond; OHT-non-OCL: orthotopic heart transplant recipients without obstructive coronary lesions on coronary computed tomography angiography; OHT-OCL: orthotopic heart transplant recipients with obstructive coronary lesions on coronary computed tomography angiography; PWd: posterior wall thickness in diastole; RWT: relative wall thickness.

**Table 3:** Strain and MW parameters in OHT-OCL patients, OHT-non-OCL patients, and HV.

	OHT-OCL (*n* = 7)	OHT-non-OCL (*n* = 48)	HV (*n* = 57)	*p*
LV-GLS, %	−10.60 (−14–−6.80)	−15.60 (−16.50–−13.40)	−18 (−20–−16)	<0.01
MWI, mmHg%	1376 (665–1574)	1389.50 (1205–1640)	1853 (1613–2064)	<0.01
GCW, mmHg%	1625 (796–1934)	1667.50 (1409–1927)	2142 (1879–2328)	<0.01
GWW, mmHg%	136 (74–145)	83.50 (62.50–118.50)	59 (38–83)	<0.01
GWE, %	87 (86–92)	94 (91–95)	96 (95–97)	<0.01

The echocardiographic findings for advanced parameters across the three study groups are shown. Values are presented as means and standard deviations for data that follow a normal distribution and as median and interquartile ranges for data not normally distributed. *p*-values refer to overall differences among the three groups. GCW: global constructive work; GWE: global work efficiency; GWW: global wasted work; HV: healthy volunteers; LV-GLS: global longitudinal strain of left ventricle; MWI: myocardial work index; OHT-non-OCL: orthotopic heart transplant recipients without obstructive coronary lesions on coronary computed tomography angiography; OHT-OCL: orthotopic heart transplant recipients with obstructive coronary lesions on coronary computed tomography angiography.

**Table 4:** Diagnostic performance of conventional and advanced echocardiographic parameters: AUC, confidence intervals, cut-off values by Youden Index, sensitivity, and specificity.

	AUC	Lower Limit CI	Upper Limit CI	Cut-Off	Sensibility	Specificity
IVSd, mm	0.63	0.41	0.84	11	0.71	0.625
PWd, mm	0.76	0.64	0.89	9.9	1	0.646
LVDd, mm	0.49	0.20	0.79	39	0.71	0.812
LV mass index, gr/m^2^	0.65	0.40	0.90	98	0.57	0.812
RWT	0.65	0.44	0.86	0.48	0.71	0.71
Biplane EDV index, ml/m^2^	0.64	0.38	0.90	34.6	0.71	0.73
Biplane ESV index, ml/m^2^	0.70	0.44	0.95	15	0.14	0.83
LV-EF, %	0.69	0.42	0.96	60.6	0.71	0.77
E wave cm/s	0.31	0.07	0.54	53	0.43	0.21
E wave DT, ms	0.50	0.20	0.79	131	0.43	0.81
E/A	0.70	0.51	0.88	1.61	1	0.19
e’ septal cm/s	0.61	0.37	0.86	59	0.43	0.85
e’ lateral cm/s	0.62	0.37	0.85	86	0.57	0.79
Mean E/e’	0.48	0.26	0.70	7.4	0.29	0.47
IRT, ms	0.54	0.32	0.76	83	0.57	0.67
LV-GLS, %	0.80	0.59	1.01	−14.4	0.86	0.71
MWI, mmHg%	0.58	0.33	0.84	772	0.29	1
GCW, mmHg%	0.57	0.29	0.85	1058	0.29	1
GWW, mmHg%	0.70	0.49	0.90	117	0.71	0.75
GWE, %	0.75	0.52	0.97	89	0.71	0.85

AUC are shown in the table above, along with the lower and upper limits of the CI, the cut-off values estimated using Youden Index, and the sensibility and specificity for each echocardiographic variable to identify patients with OCL. Values are presented as means and standard deviations for data that follow a normal distribution and as median and interquartile ranges for data not normally distributed. AUC: area under the curve; biplane EDV index: left ventricular end-diastolic volume index; cm/s: centimeters per second; LV-EF: left ventricular ejection fraction; biplane ESV index: left ventricular end-systolic volume index; E wave DT: deceleration time of E-wave; E wave: early diastolic mitral inflow velocity; E/A: ratio of E-wave to A-wave; e’ lateral: tissue Doppler E’ velocity at the lateral annulus; e’ septal: tissue Doppler E’ velocity at the septal annulus; GCW: global constructive work; GWE: global work efficiency; GWW: global wasted work; IRT: isovolumetric relaxation time; IVSd: interventricular septum thickness in diastole; lower CI: lower confidence interval; LV-GLS: global longitudinal strain of left ventricle; LV mass index: left ventricular mass index; LVDd: left ventricular end-diastolic dimension; mean E/e’: ratio of E-wave to tissue Doppler e’ velocity; ms: millisecond; MWI: myocardial work index; PWd: posterior wall thickness in diastole; RWT: relative wall thickness; upper CI: upper confidence interval.

**Table 5:** Stepwise analysis of echocardiographic parameters for OCL prediction.

Parameter	OR	95% CI	*p*	PseudoR
GWE	15.65	2.06–119.20	0.008	0.23
LV-EF	9.08	1.17–70.22	0.034	0.15

Stepwise analysis of all the parameters associated with OCL in the univariate analysis, identifying LV-EF and GWE as independent predictors of the existence of OCL. CI: confidence interval; GWE: global work efficiency; LV-EF: left ventricle ejection fraction; OR: odds ratio.

**Table 6:** Post hoc power analysis.

Comparison	GWE Power (%)	LV-GLS Power (%)
OHT-non-OCL vs. OHT-OCL	62%	41%
HV vs. OHT-OCL	99%	63%

GWE: global work efficiency; HV: healthy volunteers; LV-GLS: left ventricular global longitudinal strain; OHT-non-OCL: orthotopic heart transplant recipients without obstructive coronary lesions on coronary computed tomography angiography; OHT-OCL: orthotopic heart transplant recipients with obstructive coronary lesions on coronary computed tomography angiography.

## Data Availability

The data supporting the findings of this study are available upon request from the corresponding author. These data are not publicly available due to privacy and ethical restrictions, as they contain information that could compromise the confidentiality of research participants. Access to the data will be granted to qualified researchers upon reasonable request, ensuring compliance with applicable data protection regulations.

## Supplementary Materials

The following supporting information can be downloaded at: https://www.mdpi.com/article/10.3390/diagnostics15111352/s1 Table S1 shows the sociodemographic characteristics of the two OHT groups. Table S2 presents the results of the univariate analysis for all the echocardiographic variables dichotomized using the Youden Index in OHT patients without vs with OCL.

## Abbreviations

The following abbreviations are used in this manuscript:

A wave	late diastolic mitral inflow velocity
ANT	anterior
ANT_SEPT	anteroseptal
AUC	area under the curve
Biplane EDV index	left ventricular end-diastolic volume index
LV-EF	biplane ejection fraction; left ventricular ejection fraction
Biplane ESV index	left ventricular end-systolic volume index
BMI	body mass index
CAD	coronary artery disease
CAV	cardiac allograft vasculopathy
CCTA	coronary computed tomography angiography
CI	confidence interval
cm/s	centimeters per second
CO	cardiac output
DBP	diastolic blood pressure
DT	deceleration time
E wave	early diastolic mitral inflow velocity
E/A	ratio of E-wave to A-wave
e’ lateral	tissue Doppler E’ velocity at the lateral annulus
e’ septal	tissue Doppler E’ velocity at the septal annulus
ECMO	extracorporeal membrane oxygenation
EDS	end systolic volume
EDV	end diastolic volume
eGFR	estimated glomerular filtration rate
GCW	global constructed work
GWE	global work efficiency
GWW	global wasted work
HLA	human leukocyte antigens
HV	healthy volunteers
IABP	intra-aortic balloon pump
ICA	invasive coronary angiography
INF	inferior
IRT	isovolumetric relaxation time
ISHLT	International Society for Heart and Lung Transplantation
IVSd	interventricular septum thickness in diastole
IVUS	intravascular ultrasound
LAT	lateral
LV	left ventricle, left ventricular
LV-EF	left ventricular ejection fraction
LV-GLS	left ventricular global longitudinal strain
LV mass index	left ventricular mass index
LVDd	left ventricular end-diastolic diameter
Mean E/e’	ratio of E-wave to tissue Doppler e’ velocity
ms	millisecond
MW	myocardial work
MWI	myocardial work index
NPV	negative predictive value
OCL	obstructive coronary lesions
OHT	orthotopic heart transplantation
OHT-non-OCL	OHT patients without coronary lesions or with lesions <50% on the CCTA
OHT-OCL	OHT patients with lesions ≥50% on the CCTA
OR	odds ratio
POST	posterior
PRA	panel reactive antibodies
PWd	posterior wall thickness in diastole
ROC	receiver operating characteristic
RWT	relative wall thickness
SBP	systolic blood pressure
SEPT	septal
TTE	transthoracic echocardiography
