# Supplementary material for: Non-Invasive Myocardial Work Identifies Patients with Obstructive Coronary Lesions After Orthotopic Heart Transplantation
Source: Diagnostics (Basel). 2025 May 28;15(11):1352. doi: 10.3390/diagnostics15111352 (PMC12155252; doi:10.3390/diagnostics15111352)
Supplement: Supplementary file 1 [file diagnostics-15-01352-s001.zip › diagnostics-3647354-supplementary.pdf]

Supplementary materials
Table 1: Demographic and clinical characteristics of OHT-non-OCL and OHT-OCL patients

	OHT-non-OCL (n=48)	OHT-OCL (n=7)	p
**Age (years)**	67.52 (54.89-73.48)	65.20 (56.33-71.76)	.96
**Donor age (years)**	46.00 (32.50-53.00)	44.00 (35.00-48.00)	.81
**Donor transmitted CAD**			.60
**No CAD**	38 (79.17)	6 (85.71)	
**Non-significant CAD**	3 (6.25)	1 (14.29)	
**Significant CAD**	6 (12.50)	0	
**Sex (male)**	35 (72.92)	7 (100)	.179
**BMI (kg/m²)**	26.20 (22.90-27.85)	25.30 (21.60-29.80)	.87
**SBP (mmHg)**	126.15 (17.80)	126.14 (28.87)	.742
**DBP (mmHg)**	80.50 (12.29)	80 (17.50)	>.99
**Hypertension**	30 (62.50)	5 (71.43)	>.99
**Dyslipidemia**	33 (68.75)	6 (85.71)	.66
**Diabetes mellitus**	8 (16.67)	5 (71.43)	<.01
**Tobacco use**			.096
**Never**	23 (48.94)	1 (14.29)	
**Current**	2 (4.26)	0 (0)	

**Former**	22(46.81)	6 (85.71)	
**Age at OHT (years)**	57 (46-63)	51 (46-56)	.455
**Retransplant**	1 (2.08)	0 (0)	>.99
**eGFR (mL/min/1,73 m²)**	61 (54.50-61)	51 (37-61)	.029
**Hemoglobin (g/dl)**	13.50 (12.55-15.10)	13.90 (12.50-14.40)	.82
**Cytomegalovirus infection**	22 (45.83)	2 (28.57)	.451
**Years since OHT**	7.0 (5-12.1)	11.5 (6.2-19.9)	.198
**Cardiac device**	1 (2.08)	1 (14.29)	.24
**Peripheral artery disease**	4 (8.33)	2 (28.57)	.163
**Etiology of the cardiopathy:**			
**Dilated**	19 (39.58)	4 (57.14)	.435
**Ischemic**	18 (37.50)	3 (42.86)	>.99
**Valvular**	5 (10.42)	0 (0)	>.99
**Congenital**	4 (8.33)	0 (0)	>.99
**CAV**	1 (2.08)	0 (0)	>.99
**Autoimmune**	1 (2.08)	0 (0)	>.99
**Non-isogroup ABO**	6 (13.04)	0 (0)	.58

**Urgent code**	9 (19.57)	2 (28.57)	.63
**ECMO-bridge to transplant:**<br>**No ECMO**<br>**ECMO**<br>**ECMO + IABP**	 39 (81.25)<br>7 (14.58)<br>2 (4.17)	 5 (71.43)<br>0 (0)<br>2 (28.57)	.11
**Ischemic time (min)**	222.66 (45.56)	234.29 (43.67)	.46
**Post-transplant ECMO**	3 (6.25)	1 (14.29)	.43
**Antiplatelet:**<br>**Acetylsalicylic acid**<br>**Clopidogrel**<br>**Ticlopidine**	 38 (79.17)<br>5 (10.42)<br>4 (8.33)	 5 (71.43)<br>3 (42.86)<br>1 (14.29)	 .639<br>.055<br>.508
**Immunosuppressive agents:**<br>**Tacrolimus**<br>**Mycophenolate mofetil**<br>**Everolimus**<br>**Steroids**<br>**Cyclosporine**<br>**Sirolimus**	 45 (93.75)<br>40 (83.33)<br>8 (16.67)<br>7 (14.58)<br>2 (4.17)<br>1 (2.08)	 7 (100)<br>2 (28.57)<br>5 (71.43)<br>1(14.29)<br>0 (0)<br>0 (0)	 >.99<br><.01<br><.01<br>>.99<br>>.99<br>>.99
**Lipid-lowering drugs:**<br>**Statin**<br>**Ezetimib**	 42 (87.50)<br>5 (10.42)	 6 (85.71)<br>5 (71.43)	 >.99<br><.01

Table 1 *shows the sociodemographic characteristics of the two OHT groups. Values are presented as means and standard deviations for data that follow a normal distribution and as median and interquartile ranges for data not*

normally distributed. BMI: body mass index; CAD: coronary artery disease; DBP: diastolic blood pressure; ECMO: extracorporeal membrane oxygenation; eGFR: estimated glomerular filtration rate; HV: healthy volunteers; IABP: intra-aortic balloon pump; OHT-non-OCL: orthotopic heart transplant recipients without obstructive coronary lesions on coronary computed tomography angiography; OHT–OCL: orthotopic heart transplant recipients with obstructive coronary lesions on coronary computed tomography angiography; SBP: systolic blood pressure.

Table 2. Univariate analysis for echocardiographic variables dichotomized using the Youden Index in OHT patients without vs with OCL.

	OHT-non-OCL (n=48)	OHT-OCL (n=7)	p
**IVSd (mm)**	18 (37.50)	5 (71.43)	.116
**PWd (mm)**	17 (35.42)	7 (100)	.002
**LVDd (mm)**	39 (81.25)	5 (71.43)	.617
**LV mass index (gr/m²)**	9 (18.75)	4 (57.14)	.046
**Biplane EDV index (ml/m²)**	13 (27.08)	5 (71.43)	.032
**Biplane ESV index (ml/m²)**	8 (16.67)	4 (57.14)	.034
**LV-EF (%)**	11 (22.92)	5 (71.43)	.018
**E wave (cm/s)**	38 (79.17)	3 (42.86)	.061
**E wave DT (ms)**	9 (19.15)	3 (42.86)	.175
**E/A**	28 (59.57)	7 (100)	.044
**e′ septal (cm/s)**	7 (14.89)	3 (42.86)	.109
**Mean E/e'**	22 (46.81)	2 (28.57)	.443
**IRT (ms)**	15 (32.61)	4 (57.14)	.234

**LV-GLS (%)**	14 (29.17)	6 (85.71)	.007
**MWI (mmHg%)**	0 (0)	2 (28.57)	.014
**GCW (mmHg%)**	0 (0)	2 (28.57)	.014
**GWW (mmHg%)**	12 (25)	5 (71.43)	.024
**GWE (%)**	7 (14.58)	5 (71.43)	.004

***Table 2** presents the results of the univariate analysis for all the echocardiographic variables dichotomized using the Youden Index in OHT patients without vs with OCL. Biplane EDV index: left ventricular end-diastolic volume index; cm/s: centimeters per second; LV-EF: left ventricular ejection fraction; biplane ESV index: left ventricular end-systolic volume index; E wave DT: deceleration time of E-wave; E wave: early diastolic mitral inflow velocity; E/A: ratio of E-wave to A-wave; e′ lateral: tissue Doppler E′ velocity at the lateral annulus; e′ septal: tissue Doppler E′ velocity at the septal annulus; GCW: global constructive work; GWE: global work efficiency; GWW: global wasted work; IRT: isovolumetric relaxation time; IVSd: interventricular septum thickness in diastole; LV-GLS: global longitudinal strain of left ventricle; LV mass index: left ventricular mass index; LVDd: left ventricular end-diastolic dimension; mean E/e′: ratio of E-wave to tissue Doppler e′ velocity; ms: millisecond; MWI: myocardial work index; OHT-non-OCL: orthotopic heart transplant recipients without obstructive coronary lesions on coronary computed tomography angiography; OHT–OCL: orthotopic heart transplant recipients with obstructive coronary lesions on coronary computed tomography angiography; PWd: posterior wall thickness in diastole.*
